# Diminished antimicrobial drug use in dogs with allergic dermatitis treated with oclacitinib

**DOI:** 10.3389/fvets.2023.1207582

**Published:** 2023-09-07

**Authors:** Kennedy Mwacalimba, Andrew Hillier, Michele Rosenbaum, Christopher Brennan, Deborah Amodie

**Affiliations:** ^1^Zoetis Outcomes Research, Parsippany, NJ, United States; ^2^Zoetis Veterinary Professional Services, Parsippany, NJ, United States; ^3^Zoetis Market Research, Parsippany, NJ, United States

**Keywords:** dogs, pyoderma, oclacitinib, glucocorticoids, antimicrobial use

## Abstract

**Introduction:**

Dogs with allergic dermatitis often suffer concurrent skin and ear infections. The objective of this study was to retrospectively quantify the number of systemic and topical antimicrobial transactions in dogs with allergic dermatitis, following administration of oclacitinib or a glucocorticoid, compared to dogs that did not receive a pruritus therapy when there is an initial diagnosis of pyoderma. A secondary objective was to demonstrate that dogs on oclacitinib use fewer antimicrobials and concomitant therapies over time and have improved quality of life.

**Materials and methods:**

This was a retrospective case–control study using a large, centralized database to identify canine patients receiving pruritus therapy along with a concurrent diagnosis of pyoderma. For the second objective, 58 client-owned dogs diagnosed with allergic dermatitis were enrolled in a prospective owner and dog quality of life and treatment satisfaction (QoL&TS) study that also evaluated concomitant therapy use over time. In Part A, data consisted of anonymous transaction records from 1,134 hospitals across the United States, representing pyoderma visits between December 2018 and December 2019. Odds ratios comparing the relative odds of having additional antimicrobial agent transactions were calculated, given initial pruritus therapy compared to dogs that did not receive pruritus therapy. Parametric bootstrapping was used to calculate goodness-of-fit statistics. In part B, dogs entered the study on Day 0 and returned for examination on Days 14, 21, 30, and 60. Owner determination of QoL&TS was performed on Days 0, 1, 3, 14, 21, 30, and 60. On Days 0, 14, 21, and 60, a veterinarian assessed concomitant therapies and dermatitis severity scoring. Least Squares Means and Standard Errors for QoL&TS, and Dermatitis Vet VAS (Visual Analog Scale) Scores were calculated using a Linear Mixed Model Approach for Repeated Measures (α = 0.05). The percent reduction in therapies was also calculated.

**Results:**

Dogs that received oclacitinib (n = 5,132) or a glucocorticoid (*n* = 7,024) had reduced odds (OR: 0.8091; *p* = 0.0002 and OR: 0.7095; *p* < 0.0001, respectively) of having a follow up antimicrobial drug transaction after initial antimicrobial therapy compared to dogs with no pruritus therapy at the initial visit (*n* = 12,997). In part B, oclacitinib demonstrated a statistically significant improvement in QoL&TS scores over time QoL (*p* < 0.05). Veterinarian assessment showed a 70% reduction in dermatitis severity over time (*p* < 0.05), supporting oclacitinib’s anti-inflammatory effects. Oclacitinib therapy was also associated with an 83% reduction in concomitant treatments, including a 100% reduction in systemic antimicrobial therapy over eight weeks.

**Discussion:**

Dogs receiving oclacitinib showed no increase in antimicrobial therapy transactions compared to glucocorticoid recipients at the initial pyoderma diagnosis. Having a pruritus therapy at the index pyoderma visit reduced the odds of subsequent antimicrobial transactions. In addition to reducing concomitant therapy usage, oclacitinib improved owner and pet QoL, suggesting a paradigm shift in treatment success that could reshape allergic pruritus therapy recommendations. The study provides empirical evidence of oclacitinib’s reduction in antibacterial therapy, supporting its therapeutic value and antimicrobial stewardship.

## Introduction

Dogs with allergic or atopic dermatitis suffer concurrent skin and ear infections (1–3). Staphylococci, in particular, have increased adherence to inflamed and atopic skin (4, 5). Treatment often requires topical and/or systemic antimicrobial therapy (2, 6), but this must adhere to the principles of judicious antimicrobial use. Antimicrobial stewardship can be aided by the use of treatments that reduce the need for antimicrobials when treating allergic skin disease (6). Antimicrobial drugs are a powerful tool for both human and animal health, and for them to remain effective and sustainable, they must be used responsibly.

In the United States, oclacitinib (Apoquel^®^ tablet) is indicated for the control of pruritus associated with allergic dermatitis and control of atopic dermatitis in dogs at least 12 months of age. It may be given twice daily for up to 14 days of therapy and once daily thereafter for maintenance. Oclacitinib may be used concomitantly with many other common therapies, such as vaccines, NSAIDs, antimicrobials, and allergen immunotherapy (7). It inhibits the function of a variety of pro-inflammatory, pro-allergic, and pruritogenic cytokines that are dependent on Janus kinase (JAK) enzyme activity, selectively inhibiting JAK1 primarily and less so JAK3 (7).

Glucocorticoids are among the most widely used chemotherapeutic agents in veterinary medicine (8). A study in the United Kingdom, for example, demonstrated that 20% of skin cases involved the prescription of a systemic glucocorticoid (9), and dogs diagnosed with dermatitis had increased odds of receiving glucocorticoid therapy. However, glucocorticoid receptors are ubiquitous in most cells, and this creates the potential for a multitude of intended and unintended effects when the agents are administered as therapies (10). Long-term glucocorticoid therapy is ill-advised in the management of canine pruritus due to the potential for serious adverse effects (11). Glucocorticoids remain highly effective and safe for the management of allergic skin disease when used appropriately (12).

Anonymized primary-care veterinary practice records offer potential for investigating various aspects of therapy and health outcomes (13). The current study aimed to compare the frequency of antibiotic purchases recorded in the electronic transaction records of a large population of dogs with a diagnosis of pyoderma under primary veterinary care in the United States. The study further aimed to evaluate antibiotic and concomitant therapy usage in dogs treated with oclacitinib.

This observational study had two objectives:

1. To retrospectively quantify the number of subsequent systemic and topical antimicrobial transactions in client-owned dogs with allergic dermatitis, including atopic dermatitis, following administration of oclacitinib, glucocorticoids, or other pruritus therapy compared to dogs that did not receive a pruritus therapy when there is an initial diagnosis of pyoderma.
2. To demonstrate that dogs that are on oclacitinib use fewer concomitant therapies, including antimicrobials, over time.

## Materials and methods

### Objective 1

For objective 1, a large, centralized database (Covetrus Inc., Portland, ME) was used to identify canine patients that had received a pruritus therapy along with a concurrent diagnosis of pyoderma, using a case control approach. Transaction records were anonymous and represented hospitals across the United States, looking only at visits mentioning pyoderma and had product combination purchases that strongly suggested the management of allergic or atopic dermatitis during the time between December 2018 and December 2019. Review of data followed the pharmacoeconomic guidelines and checklist for compliance and persistence studies using retrospective databases (14). Covetrus provided anonymized transaction data from practices that met the study inclusion criteria defined below:

1. Primary cases: oclacitinib was prescribed for the treatment of pruritus associated with allergic dermatitis or a clinical presentation of atopic dermatitis in dogs aged ≥12 months.
2. Secondary cases: a systemic glucocorticoid, antihistamine, or other anti-pruritic therapy was prescribed for the treatment of pruritus associated with atopic or allergic dermatitis or a clinical manifestation of atopic dermatitis in dogs aged ≥12 months.
3. Controls: pyoderma managed without an antipruritic therapy administered at the index visit

The database was queried for records of patients with confirmed purchases of pruritus products (oclacitinib, glucocorticoid, antihistamine, or other).

- The dog is at least 12 months old and presenting with itching
- The dog is diagnosed with allergic dermatitis or allergic itch (+ pyoderma) on record

The following inclusion criteria were used to identify canine pruritus visits from the transaction records:

- Record of oclacitinib, glucocorticoids (systemic or topical), cyclosporine, or antihistamines, purchased in combination with a topical shampoo, topical antimicrobial, systemic antimicrobial, and/or dermatologic/hypoallergenic diet, and/or Flea/Tick product
- Provision of services like cytology/skin cytology/ear cytology scraping/skin scraping
- Antimicrobial agents most prescribed for bacterial skin infections such as cephalexin, cefpodoxime, cefovecin sodium (Convenia^®^), amoxicillin, and clavulanate potassium and no other evidence of this antimicrobial agent being used for a non-skin infection

The following criteria were applied to the search query:

### Index visit

- Look for transactions explicitly mentioning pyoderma between Dec 2018 and Dec 2019
- Exclude patients with standalone antipruritic, glucocorticoid, or antihistamine purchases 30 days prior to the first pyoderma visit to ensure that this was an index pyoderma visit
- Exclude patients with antimicrobial agent purchases 30 days prior to the first pyoderma visit
- Consider 3 days after the initial pyoderma visit as one treatment and look for antifungal, antihistamine, antipruritic agent, glucocorticoid, and/or antimicrobials purchases

### Look forward

- Look forward 30 days from the index pyoderma visit
- Capture whether the patient had an antimicrobial purchase and the number of visits with antimicrobials transactions
- Capture what type of visit patient had in the subsequent 30 days and tag in the following priority order
- Pyoderma Visit – visit with mention of pyoderma
- Dermatologic/Antimicrobial agent Visit – visit with purchase of antimicrobials, pruritus products or skin test
- Exam Visit – visit with any exam
- Unknown Visit – any visit
- Capture number of days between the initial pyoderma visit and next visit defined in the priority order above

Each patient was then tracked for repeat visits in which pyoderma remained a concurrent finding within 14, 21, or 30 days of the index visit with a look forward period of up to 6 months. The database was reviewed for evidence of probable resolution (e.g., no antimicrobial agent dispensed on follow up visit and/or no pyoderma mentioned in record on follow up visit), extension of/or switch in antimicrobial agent therapy (different antimicrobial agent dispensed/pyoderma mentioned in follow up visit), and recurrence, i.e., dogs with pyoderma or antimicrobial agents dispensed after 30 days of the index visit (Table 1).

**Table 1:** Database query search terms and guidelines.

*Primary search*
Bacterial Pyoderma; Superficial Pyoderma; Cutaneous Pustule; Pustule; Pustule of Skin; Skin Pustule; Superficial Pustule; hot spot Skin cytology done and bacteria recorded in medical record
*Secondary search*
Suspected allergic dermatitis with secondary Staphylococcal pyoderma: allergic dermatitis or allergic itch; Acute flare of atopic dermatitis
*Antimicrobial agent class administered*
Aminopenicillin, Cephalosporin, Fluoroquinolone, Lincosamide, Sulfonamide No other evidence of this antimicrobial agent being used for a non-skin infection
*Concurrent pruritus therapies*
Oclacitinib Systemic glucocorticoids Antihistamines Neither Oclacitinib nor glucocorticoids (i.e., other)
*Look forward period after identification of index visit*
Track for repeat visits in which pyoderma is a concurrent finding within 14, 21, or 30 days of index visit and length of antimicrobial agent therapies (e.g., cephalexin 14 days, cefovecin sodium 14 days/1 administration etc) Review medical record for Evidence of resolution/improvement (no antimicrobial agent dispensed on follow up visit and/or no pyoderma mentioned in record or negative cytology) Extension of/or switch in antimicrobial agent therapy

### Objective 2

Client-owned dogs diagnosed with allergic or atopic dermatitis were enrolled in a prospective open label, non-randomized, non-blinded quality of life (QoL) and treatment satisfaction study. This study centered on using oclacitinib as the primary treatment for itching. Conducted across four U.S. veterinary clinics, the study included dogs with moderate to severe itching and a history of allergic (flea, food, contact allergy or allergic dermatitis of undetermined cause) or atopic dermatitis (environmental allergy), either individually or combined as determined by the attending veterinarian. Dogs with concurrent non-cutaneous diseases were eligible if their treatment remained stable for 6 weeks before the study with no expected changes during the study. Flea-free status and prescription flea control/prevention throughout the study were required. Exclusions comprised dogs with unrelated ill-health on Day 0, severe infections, breeding intent, pregnant/lactating status, malignant disorders, progressive malignancies, and evidence of immune suppression (e.g., hyperadrenocorticism, demodicosis) on Day 0. Veterinarians were advised to report any treatment failures or adverse events associated with the therapy.

Dogs entered the study on Day 0 and returned for examination on Days 14, 21, 30, and 60. Owner determination of QoL and Treatment Satisfaction (TS) assessment was performed on Day 0, 1, 3, 14, 21, 30, and 60 using a Canine Dermatitis Quality of Life and Treatment Satisfaction Questionnaire, which was developed based on owner experience of caring for their dog with atopic or allergic dermatitis (15) and validated according to FDA guidelines (16). Oclacitinib was dosed at 0.4 to 0.6 mg/kg twice daily (BID) for 14 days followed by once daily (SID) dosing. The primary variables of interest were dog QoL, owner QoL, Treatment Satisfaction, and Veterinarian Assessment of Dermatitis Visual Analog Scale scores. A quantitative assessment of concurrent medications administered during the observation period was also performed.

### Data analysis

For objective 1, outputs were determined from an analysis of raw data associated with dogs with a diagnosis of pyoderma at the index visit. *A priori* outputs were additional anti-infective (antimicrobial agent) transactions after the index visit. The data were anonymized, summarized, and entered in Microsoft Excel™. Data analysis compared the relative odds of having an antimicrobial agent transaction, given an initial pruritus therapy at index visit compared to dogs that did not receive a pruritus therapy. Odds ratios, standard error, 95% confidence intervals (α = 0.05), and associated *p*-values were determined according to Sheskin (17).

To further determine if there was a difference in the number of antimicrobial agent transactions for dogs receiving oclacitinib compared to those receiving glucocorticoids, the parametric bootstrapping application in @Risk (Palisade Corporation) was used to calculate parameter confidence intervals and goodness-of-fit statistics. All available data were used to calculate the average number of visits within the 30-day observation window. The parametric bootstrapping calculation assumed that the transaction data came from a known distribution with unknown parameters (18). Ten thousand bootstrapping simulations were run for oclacitinib, glucocorticoid, and no pruritus therapy transactions.

For objective 2, primary variables were analyzed by a Generalized Linear Mixed Model (GLMM) approach for repeated measures. Comparisons of Least Square Means were performed by the two-sided Student’s t-test at 5% level of significance. A percent reduction in therapies was calculated based on concomitant therapy assessments performed on Days 0, 14, 21, and 60.

## Results

For objective 1, data were from a sample of 1,134 practices and a population of 47,856 canine patients. Every dog in the study had transactions aligning with the management of allergic dermatitis, and explicit mentions of pyoderma in their record. However, the absence of structured diagnostic codes and inconsistencies in data entry across the 1,134 practices’ practice management systems prevented us from determining specific pyoderma types (superficial or deep) with the desired level of clinical detail. In total, 22% of patients (*n* = 10,233) with a diagnosis consistent with pyoderma received a glucocorticoid, 16% (*n* = 7,646) received oclacitinib, and 43% of dogs received no antipruritic therapy at the index visit (*n* = 20,419). Looking only at patients that received an anti-microbial therapy at the index visit, dogs prescribed oclacitinib (*n* = 5,132) had reduced odds (OR: 0.8091, 95% CI: 0.7228–0.9056, *p* = 0.0002) of having a follow up antimicrobial agent transaction after the initial antimicrobial agent therapy compared to 12,997 dogs with no anti-pruritic therapy at the initial visit. Dogs receiving glucocorticoids (*n* = 7,024) also had reduced odds (OR: 0.7095, 95% CI 0.6391–0.7878, *p* < 0.0001) of having a follow up antimicrobial agent transaction after the initial antimicrobial agent transaction compared to dogs with no anti-pruritic therapy at the index visit.

Dogs (n = 2,531) receiving anti-pruritic therapies other than oclacitinib or glucocorticoids had increased odds of having additional antimicrobial agent transactions within 30 days compared to dogs that had no pruritus therapies (OR: 1.6538, 95% CI 1.3816–1.9796, *p* < 0.0001). Dogs prescribed an antifungal medication (*n* = 600) had increased odds (1.9105) of having additional antimicrobial agent transactions within 30 days compared to dogs that had no pruritus therapies (95% CI 1.5409–2.3688, *p* < 0.0001). There was no statistical difference in the odds of dogs prescribed antihistamines (*n* = 355; OR: 1.0621) having additional antimicrobial agent transactions within 30 days compared to dogs that had no pruritus therapies (*p* = 0.7262). When all anti-pruritic therapy patients (*n* = 18,492) were compared to non-pruritus therapy patients, receiving pruritus therapy reduced the odds of having additional antimicrobial agent visits in the subsequent 30 days (OR: 0.8899, 95% CI 0.8257–0.9592, *p* = 0.0023; Table 2).

**Table 2:** Comparison of the relative odds of having an additional antimicrobial agent visit by index visit treatment modality.

Group	Antimicrobial therapy transaction at index visit	Additional antimicrobial agent	No subsequent antimicrobial agent	Odds Ratio	95% CI	*p* value	Comment
Oclacitinib	5,132	441	4,691	0.8091	0.7228–0.9056	*p* = 0.0002	Sig.
Other	974	157	817	1.6538	1.3816–1.9796	*p* < 0.0001	Sig.
Glucocorticoid	7,024	535	6,489	0.7095	0.6391–0.7878	*p* < 0.0001	Sig.
All Other	2,531	285	2,246	1.1074	0.9677–1.2672	*p* = 0.1381	Not sig.
Antihistamine	355	39	316	1.0621	0.7580–1.4884	*p* = 0.7262	Not sig.
Antifungal	600	109	491	1.9105	1.5409–2.3688	*p* < 0.0001	Sig.
All Pruritus	18,492	1,733	16,759	0.8899	0.8257–0.9592	*p* = 0.0023	Sig.
No Pruritus Therapy	12,997	1,353	11,644	Referent	NA	NA	NA

There was no statistical difference in the odds of having additional antimicrobial agent transactions for dogs receiving oclacitinib compared to glucocorticoids (OR: 1.1402; 95% CI: 0.9997–1.3005 *p* > 0.05). There was reduced odds of having additional antimicrobial agent transactions for dogs receiving oclacitinib compared to dogs getting antifungal medication (OR: 0.4235; 95% CI 0.3367–0.5326; *p* = 0.0001).

To determine whether dogs prescribed oclacitinib were on average likely to have more antimicrobial agent transactions compared to dogs receiving glucocorticoids in the 30 days after the index visit, all the antimicrobial agent transaction data was modeled using @Risk. The parametric bootstrapping application in @Risk (Palisade Corporation), was used to calculate parameter confidence intervals and goodness-of-fit statistics for antimicrobial agent transactions for dogs receiving oclacitinib, glucocorticoids or no pruritus therapy using all the available data.

The parametric bootstrapping calculation assumed that the antimicrobial agent transaction data came from a known distribution with unknown parameters. In total, 10,000 bootstrapping simulations were run. The models that best describe the data suggest that dogs getting a glucocorticoid are likely to have a mean of 1.67 antimicrobial agent visits in the 30-day observation window (maximum of 5.01 antimicrobial agent visits, std. deviation 1.18 antimicrobial agent visits) (Table 3) compared to a mean of 1.34 visits for oclacitinib (maximum of 4.02 associated with an antimicrobial agent transaction in 30 days, std. deviation 0.947 antimicrobial agent visits) and a mean 2.0 antimicrobial agent visits for no pruritus therapy dogs (maximum of 4.01 visits within 30 days, std. deviation 1.13 visits). Therefore, dogs receiving no pruritic therapy had the greatest number of mean antimicrobial agent visits (2.0) followed by dogs receiving glucocorticoids (1.67), while oclacitinib dogs had a mean antimicrobial agent visit count of 1.34.

**Table 3:** Parametric bootstrap output best fit distribution comparing number of antimicrobial agent visits for dogs receiving oclacitinib, glucocorticoids, or no pruritus therapy at the index visit.

Treatment	Oclacitinib	Glucocorticoid	No dermatologic therapy
	Antimicrobial agent transaction count	Antimicrobial agent transaction count	Antimicrobial agent transaction count
Best Distribution Fit	Triangular	Triangular	Uniform
Maximum	4.0178	5.0105	4.005
Mean	1.3393	1.6702	2
Median	1.1768	1.4675	2
Std. Deviation	0.947	1.181	1.1576

For objective 2, data were from 58 dogs enrolled in a study to determine the quality of life and treatment satisfaction benefits of oclacitinib over a 2-month observation period. The mean age of the dogs was 6.22 years: the lowest was 1 and the highest was 14.00. The mean weight was 45.69 lbs.: the lowest weight was 8.00 lbs. and the highest was 127.00 lbs. A gender summary of patients is provided in Table 4. Out of the enrolled dogs, four were administered an oral glucocorticoid before the study began (Day 0) and on Day 14 at the discretion of the attending veterinarian. Additionally, three dogs received a glucocorticoid on Day 21, six on Day 30, and five on Day 60. Of these, only two dogs received multiple oral glucocorticoid treatments during the 60-day observation period. Therefore, no comparisons of the QoL impact of glucocorticoids vs. oclacitinib could be made.

**Table 4:** Demographic data summary.

Sex	Count
FS	23
F	6
MN	26
M	3

Least Squares Means and Standard Errors for Dog QoL, Owner QoL, Treatment Satisfaction, and Dermatitis Vet VAS (Visual Analog Scale) Scores were calculated using a Linear Mixed Model Approach for Repeated Measures (*α* = 0.05). In this study, compared to baseline, dogs on oclacitinib showed significant improvements in Dog QoL, owner QoL, Treatment Satisfaction, and veterinarian reported dermatitis severity scores (Table 5). The repeated measures analysis demonstrated significant variations in Dog QoL across different time points (*p* = 0.0001). The multiple comparison of LSMeans for time indicated notably higher dog QoL scores on Days 14, 21, and 60 compared to Day 0.

**Table 5:** Summary results of study variables (LS means and Standard Error).

Variable	LSMean ± Standard Error						
	Day 0	Day 1	Day 3	Day 14	Day 21	Day 30	Day 60
DOG QoL	77.80^c^ ± 6.18	76.93^c^ ± 6.18	77.57^c^ ± 6.18	82.31^ab^ ± 6.18	83.10^ab^ ± 6.18	80.20^bc^ ± 6.19	85.41^a^ ± 6.22
OWNER QoL	39.36^c^ ± 6.37	44.12^c^ ± 6.39	52.83^b^ ± 6.39	65.77^a^ ± 6.40	70.46^a^ ± 6.39	67.77^a^ ± 6.41	72.51^a^ ± 6.47
Treatment Satisfaction (Oclacitinib)	69.14^c^ ± 3.34	74.01^b^ ± 2.57	76.78^ab^ ± 2.53	79.32^a^ ± 2.46	80.11^a^ ± 2.51	77.05^ab^ ± 2.51	79.12^ab^ ± 2.65
Vet Reported Dermatitis VAS	67.15^a^ ± 5.64	NA	NA	29.12^b^ ± 5.81	24.6^bc^ ± 5.88	21.62^c^ ± 5.87	20.08^c^ ± 5.90

Where letter superscripts on LSMeans are the same in each row, this indicates no statistical difference.

Similarly, the repeated measures analysis indicated significant changes in Owner QoL over time (*p* = 0.0001). The subsequent multiple comparison of LSMeans for time highlighted substantial increases in owner QoL scores on Days 3, 14, 21, 30, and 60, as compared to Day 0. Additionally, scores on Days 14, 21, 30, and 60 were significantly higher when compared to those on Day 3.

The repeated measures analysis also revealed significant temporal differences in Treatment Satisfaction (*p* = 0.0001). According to the multiple comparison of LSMeans, satisfaction scores for oclacitinib treatment were notably elevated on Days 3, 14, 21, 30, and 60, in contrast to Day 0. Moreover, scores on Days 14, 21, and 60 demonstrated a considerable increase compared to Day 1.

Finally, the repeated measures analysis indicated significant variations in dermatitis VAS scores as assessed by veterinarians (*p* = 0.0001). The subsequent multiple comparison of LSMeans demonstrated that dogs exhibited significantly greater dermatitis severity on Day 0 in comparison to all other days. A substantial reduction in dermatitis severity was observed on Day 30 compared to preceding days. Notably, Day 30 and Day 60 exhibited significantly reduced dermatitis compared to Days 0 and 14. Post the initiation of oclacitinib therapy on Day 0, veterinarians recorded a 57% decrease in dermatitis severity by Day 14. Between Day 0 and Day 60, this reduction further increased to 70%. Evidently, lesion improvement was rapid post-treatment initiation and continued to progress throughout the study period from a clinical perspective.

### Concomitant therapy usage for the management of pruritus and associated lesions

All concomitant therapies the dogs enrolled in this study received prior to commencing oclacitinib therapy were recorded (Table 6; Day 0) as well as what they went home with on Days 14, 21, 30, and 60. No data was collected on how long dogs had been on these previous treatments prior to commencing oclacitinib. In total, 83% fewer concomitant dermatologic therapies were provided after 60 days on oclacitinib, i.e., from a list of 70 dermatological therapies on Day 0 down to 12 on Day 60 (Table 6). Specifically, there were 94% fewer topical dermatologic therapies prescribed (from 33 on Day 0 to 2 on Day 60), and 68% fewer systemic dermatologic therapies (from 37 on Day 0 to 7 on Day 60). Furthermore, there was 100% reduction in systemic antimicrobials associated with dermatologic care (21 systemic antimicrobial agents were prescribed on Day 0 and none on Day 60 of the study). There was an 88% reduction of topical antimicrobial agents as well (Table 6).

**Table 6:** Concomitant therapies administered alongside oclacitinib in an 8-week quality of life and treatment satisfaction study.

Day 0		Day 14		Day 21		Day 30		Day 60		% Reduction	Antibiotic reduction
Therapy	*n*	Therapy	*n*	Therapy	*n*	Therapy	*n*	Therapy	*n*		
Hypoallergenic diet	3	Systemic antibacterial	4	Systemic antibacterial	1	Oral steroid	6	Oral steroid	5		100%
Antifungal	1	Oral steroid	4	Oral steroid	3			Cytopoint	7		
Antihistamines	4	Antihistamines	1								
Miscellaneous antipruritic compounds	3										
Oral steroid	4										
Systemic antimicrobial	19										
Cefpodoxime proxetil	1										
Cephalexin	1										
Ultamino	1										
*Systemic therapy sub total*	37		9		4		6		12	68%	
Osurnia bilaterally	1	Medicated shampoos	4			Medicated shampoos	1	Topical antibacterial	2		88%
Medicated shampoos	9	Topical antibacterial	2			Colloidal oatmeal soaks	1				
Colloidal oatmeal soaks	1	Ear drops/Ophthalmic products	1			Topical antibacterial	1				
Benzoyl peroxide cream	1	TrizUltra+Keto Ear Cleaner	1								
Ear drops/Ophthalmic products	4										
Topical antibacterial	15										
Easotic	1										
NeoPredef with Tetracaine Powder	1										
Topical Therapy Subtotal	33		8				3		2	94%	
All dermatologic Total	70		17		4		9		12	83%	
Vitamins	1	Thyroid supplementation	1	Selegiline/Fluoxetine	1	Systemic NSAIDS	2	Thyroid supplementation	1		
Thyroid supplementation	1	Selegiline/Fluoxetine	1	Systemic NSAIDS	2	Phenylpropanolamine	1	Nutraceuticals	1		
Systemic NSAIDS	4	Systemic NSAIDS	1	Nutraceuticals	1	Nutraceuticals	1				
Phenylpropanolamine	1	Antidiarrheal	1								
Nutraceuticals	1										
Other Subtotal	8		4		4		4		2	75%	
Simparica Trio	1	Heartgard	1	Nexgard	1	ProHeart	1	Heartgard	1		
Heartgard	2			Frontline Gold	1						
Nexgard	2			Heartgard	1						
bordetella vaccine oral	1										
Adequan	1										
Zylkene	1										
Other Subtotal	8		1		3		1		1	88%	
Grand total	78		18		7		10		13	83%	

## Discussion

In this study, 22% of patients with a diagnosis consistent with pyoderma received a glucocorticoid, similar to findings in the study by Hill et al. (9). Dogs that were prescribed oclacitinib (*n* = 5,132) or a glucocorticoid (*n* = 7,024) concurrently with an antimicrobial therapy at the initial pyoderma visit had reduced odds (OR: 0.8091; *p* = 0.0002 and OR: 0.7095; *p* < 0.0001, respectively) of having a follow-up antimicrobial drug transaction after initial antimicrobial therapy compared to 12,997 dogs with no pruritus therapy prescribed at the initial pyoderma visit. The 355 dogs prescribed antihistamines had the same odds (OR: 1.0621) of having additional antimicrobial transactions within 30 days compared to dogs that had no pruritus therapies (*p* = 0.7262). Interestingly, 2,531 dogs receiving other dermatological therapies had increased odds (OR: 1.6538; *p* < 0.0001) of having additional antimicrobial therapy transactions within 30 days. When all pruritus therapy patients (*n* = 18,492) were compared to non-pruritus therapy patients, receiving pruritus therapy reduced the odds of having additional antimicrobial therapy visits in the subsequent 30 days (OR: 0.8899; *p* = 0.0023).

Looking at the dogs with antimicrobial therapy transactions, the parametric bootstrapping model showed slightly more antimicrobial therapy visits in dogs getting a glucocorticoid (mean 1.67) compared to oclacitinib (mean 1.34). This suggests that dogs receiving oclacitinib are likely to have fewer antimicrobial therapy transactions compared to dogs receiving glucocorticoids in the 30 days after a concurrent diagnosis of pyoderma at the index visit. While the current study was not designed to compare antimicrobial therapy selection associated with each antipruritic choice, a retrospective case–control study conducted in Australia demonstrated that use of oclacitinib to treat allergic dermatitis in dogs (*n* = 58) was associated with less amoxicillin clavulanic acid and topical neomycin–this usage was statistically significantly lower (*p* = 0.024) in oclacitinib-treated dogs vs. controls (*n* = 205), which included glucocorticoids (6). Other studies have demonstrated that there is no evidence that oclacitinib increases the frequency of skin infections in dogs with allergic dermatitis (7, 19, 20).

In the first part of this study, dogs receiving oclacitinib did not have additional antimicrobial therapy transactions compared to glucocorticoids when administered in cases where there is an initial diagnosis of pyoderma. There is no difference in the odds of having an antimicrobial therapy between the two treatment groups. Providing pruritus therapy at the index visit reduces the odds of having follow up antimicrobial therapy transactions. When modeled, the model that best described the data showed that dogs receiving oclacitinib are, on average, likely to have fewer antimicrobial transactions compared to dogs receiving glucocorticoids in the 30 days after a concurrent diagnosis of pyoderma at the index visit.

In the second part of the study, dogs receiving oclacitinib had a marked (83%) reduction in concomitant therapy administration. Noteworthy was the 100% reduction in systemic antimicrobial therapy over the 8-week observation period. Use of oclacitinib to treat allergic dermatitis in dogs was associated with less antibacterial use. Additionally, all three QoL & TS domains displayed statistically significant changes over time, with the most pronounced impact of oclacitinib therapy being observed in owner QoL. Recognizing the influence of allergic pruritic disease on owners, veterinarians may be prompted to initiate effective treatments earlier in the diagnostic process for dogs with allergic dermatitis, especially considering the emerging evidence connecting human emotional well-being, pet health outcomes, and the human–animal bond. Oclacitinib also exhibited a substantial clinical influence on the improvement of skin lesions across all timepoints, evident in the LSMeans veterinarian assessment of dermatitis severity VAS scores. These scores decreased from 67.15 on Day 0 to 20.08 on Day 60. A noteworthy 70% reduction in dermatitis severity was achieved by the study’s conclusion, underscoring the anti-inflammatory effects of oclacitinib as a primary therapy.

The assessment of treatment efficacy over time should remain intertwined with pet owners’ perceptions of improvements in their own and their pet’s QoL. Thus, beyond treatment effectiveness, veterinarians must factor in the influence of treatments on both owner and pet QoL. This expanded perspective on treatment success signifies a paradigm shift that has the potential to alter the basis for recommending therapies to manage allergic pruritus.

This study possesses certain limitations that warrant consideration. Firstly, the study’s focus on retrospective transactional data in the first part introduces potential bias in the assessment of antibiotic administration in dogs treated for allergic or atopic dermatitis. These include selection bias due to incomplete representation, as transactional data may not capture the entire population of dogs with allergic or atopic dermatitis; information bias stemming from inadequate clinical details, such as absence of accurate diagnoses, severity of conditions, and specific treatment protocols; and confounding bias from unaccounted variables in the transactional data that are associated with health outcomes and antibiotic usage which could confound the observed associations, leading to inaccurate conclusions. Additionally, the timing of data collection in transactional records might not align with the true sequence of events, affecting the accuracy of observed health outcomes and antibiotic usage patterns. However, upon systematic analysis using a Pharmacoeconomics framework (14), these data present valuable insights into potential health outcomes. This sheds light on the broader therapeutic value within the veterinary care context.

The study’s second part enhances granularity in tracking health outcomes. However, we acknowledge that the absence of a direct comparison with glucocorticoid treatment in this section restricts its scope. While glucocorticoids have demonstrated efficacy in pruritus management for dogs over many years, our primary focus was on assessing the positive influence of oclacitinib on QoL, coupled with a reduction in the use of multi-modal therapies over time, particularly in the context of antimicrobial stewardship.

Although our findings align with clinical expectations regarding reduced antimicrobial usage in dermatologic care, our study provides empirical evidence that substantiates and validates these clinical expectations. This validation is noteworthy as we examined two therapies that represent the standard of care. We conclude, therefore, that use of oclacitinib to treat allergic dermatitis in dogs is associated with less antibacterial use.

## Data availability statement

The raw data supporting the conclusions of this article will be made available by the authors, without undue reservation.

## Ethics statement

The animal studies were approved by the study underwent a Zoetis internal ethics review. Additionally, client owned dogs were in the care of their veterinarian, and informed consent was obtained to participate in the study. The studies were conducted in accordance with the local legislation and institutional requirements. Written informed consent was obtained from the owners for the participation of their animals in this study.

## Author contributions

KM: conceptualization, methodology, summary statistics, and original draft preparation. AH and MR: conceptualization and manuscript review. CB: raw data summary (Covetrus). DA: formal analysis and manuscript review. All authors approved the content of this manuscript. All authors contributed to the article and approved the submitted version.

## Funding

This research was funded by Zoetis Inc.

## Conflict of interest

KM, AH, MR, CB, and DA were employed by Zoetis Inc.

## Publisher’s note

All claims expressed in this article are solely those of the authors and do not necessarily represent those of their affiliated organizations, or those of the publisher, the editors and the reviewers. Any product that may be evaluated in this article, or claim that may be made by its manufacturer, is not guaranteed or endorsed by the publisher.
